# Low dose of lenalidmide and PI3K/mTOR inhibitor trigger synergistic cytoxicity in activated B cell-like subtype of diffuse large B cell lymphoma

**DOI:** 10.1186/s13046-016-0327-x

**Published:** 2016-03-24

**Authors:** Zhen Jin, Kai Qing, Yuan Ouyang, Zhao Liu, Wenfang Wang, Xiaoyang Li, Zizhen Xu, Junmin Li

**Affiliations:** Shanghai Institute of Hematology, State Key Laboratory for Medical Genomics, Ruijin Hospital affiliated to School of Medicine, Shanghai Jiao Tong University, Shanghai, China; Department of Hematology, Shanghai Institute of Hematology, Ruijin Hospital affiliated to School of Medicine, Shanghai Jiao Tong University, Shanghai, China; Department of Laboratory Medicine, Ruijin Hospital affiliated to School of Medicine, Shanghai Jiao Tong University, 197 Rui Jin Er Road, Shanghai, China

**Keywords:** Diffuse large B cell lymphoma, Lenalidomide, PI3K/mTOR inhibitor, Apoptosis, Cell cycle, NF-κB

## Abstract

**Background:**

Activated B cell-like subtype of diffuse large B cell lymphoma (ABC-DLBCL) presents aggressive clinical courses and poor prognosis. Targeting key pathways may raise the possibility of improving clinical outcomes.

**Methods:**

The synergetic effects were assessed by CCK-8 assay and measured by isobologram analysis. The NVP-Bez235 and lenalidomide cytotoxicity were measured by flow cytometry, Western Blot and si-RNA transfection. The combined treatment inducing tumor regression in vivo was performed in nude mice of OCI-Ly10 xenograft mouse model.

**Results:**

Low dose of two agents represented significant inhibition of proliferation with CI value < 1. NVP-Bez235 combined with lenalidomide remarkably increased apoptosis through intrinsic pathway by upregulating Bim, Bax and downregulating Bcl-xL. Akt, especially NF-κB, played an important role in the synergetic effects. Cotreatment also induced the cell cycle to be arrested in G0/G1 phase, and decreased S phase by increasing p21 expression, downregulating cyclinA and diminishing CDK2 phosphorylation in Su-DHL2 and OCI-Ly3 but not in OCI-Ly10. Mice treated with NVP-Bez235/lenalidomide represented obvious tumor growth regression and prolonged overall survival.

**Conclusions:**

Our findings demonstrated the synergistic effect of low dose of NVP-Bez235 and lenalidomide in ABC-DLBCL, the underlying mechanism may be multifunctional, involving apoptosis, Akt and NF-κB inactivation and cell cycle arrest. Cotreatment was also effective in vivo. These data pave the way for potential treatment of ABC-DLBCL with combination of NVP-Bez235 and lenalidomide.

## Background

DLBCL is the most common B-cell non-Hodgkin’s lymphoma (NHL), accounting for about 30–35 % of adults NHL cases [1]. Nearly up to one-third patients suffered from relapsed and refractory disease after R-CHOP (rituximab, cyclophosphamide, doxorubicin, vincristine, prednisolone) treatment [2], and limited patients got benefits from the salvage strategy of autologous stem cell transplantation (ASCT), indicating new agents are required to be tested in this group of people.

However, the heterogeneity made DLBCL a complex disease to handle with. DLBCL is considered to be characterize into three major subtypes with distinct activating molecular pathways and different clinical outcomes based on the gene expression profiling results [3]. Patients with activated B cell-like (ABC) subtype, characterised as constitutive NF-κB activation, were considered to have lower progression free survival (PFS) rate and overall survival (OS) rate compared with those with germinal center B-cell (GCB) subtype or primary mediastinal B-cell lymphoma (PMBL) [4]. Regarding of the participation of NF-κB in many cell processes like tumor proliferation, invasion and inflammation, targeting NF-κB pathway has become one of the promising therapeutic approach in ABC-DLBCL treatment [5]. Directly interrupting NF-κB pathway is one option of therapeutic strategy, and lenalidomide, which is used as the immunomodulatory agent treating multiple myeloma (MM), was proved to have anti-tumor effects through downregulating IRF-4 in ABC-DLBCL cell lines [6]. The selective anti-tumor effect of lenalidomide in DLBCL is also supported by clinical studies. Increasing evidence revealed that ABC-DLBCL patients got better response with higher remission rate and PFS rate when treated with lenalidomide alone or combination with R-CHOP regime [7, 8]. Meanwhile, lenalidomide was found to have inhibiting function of tumor growth through PI3K/Akt pathway, cereblon/p21, cyclin D1/p27 and Bcl-2 in chronic lymphocytic leukemia (CLL) and mantle cell lymphoma (MCL) in pre-clinical experiments [9–13]. Besides, it also showed effeciency when combined with rituximab, dexamethasone and bortezomib in clinical trials of treating several types of B-cell NHL [14, 15], which implied the potential combination with other agents in lymphoid hematological maliganancies.

Indirectly, interrupting the upstream of NF-κB could also induce the inhibition of NF-κB pathway. It is known that the constitutive NF-κB activation in ABC-DLBCL is caused by individual or overlapping known mutations like *MYD88 L265P, CARD11, TNFAIP3 and CD79B/A* [16], which are involved in antigen-specific B-cell receptor (BCR) and Toll-like receptor (TLR) induced NF-κB activation. In the signaling cascade triggered by BCR, several tyrosine kinases including PI3K, Bruton tyrosine kinase (BTK) and mTOR are participated in, subsequently inducing the downstream pathways associated with survival. NVP-Bez235 is one of the dual PI3K/mTOR inhibitors that can suppress the activity of PI3K, mTOR1 and mTOR2. It has shown anti-tumor activity in a range of hematological malignancies including MM, MCL, follicular lymphoma (FL), chronic lymphocytic leukemia (ALL) and acute myelocytic leukemia (AML) in the pre-clinical studies [17–21]. It was also reported to synergize with agents such as MEK1/2 inhibitor [22]. Inhibition of mTOR could consequently decrease the phosphorylation of P70S6 kinase as well as eukaryotic translation initiation factor 4E binding protein 1 (4EBP1), while PI3K activity represented an inexplicit relationship with mTOR in the complex cell signaling circuits.

Collectively, the findings make us to explore the efficacy of combined lenalidomide with NVP-Bez235 in treating ABC-DLBCL in vitro and in vivo. The aim of the present study was to determine whether lenalidomide could enhance the cytotoxic potency of NVP-Bez235 in ABC subtype of DLBCL, and to further elucidate the underlying mechanisms involved in this effect.

## Methods

### Cells and cell culture

The non-GCB DLBCL derived cell lines OCI-Ly10, OCI-Ly3 and Su-DHL2 were obtained from Dr. T Zhao (Nanfang Hospital affiliated to Southern Medical University, China). Cell lines were cultured in IMDM (Invitrogen, Carlsbad, USA) with 10 % FBS (Invitrogen, Carlsbad, USA), incubating in 37 °C with 5 % CO_2_.

### Apoptosis assays

Cell apoptosis was determined by flow cytometry according to the protocol of FITC Annexin V Apoptosis Detection Kit I (BD Bioscience, SanJose, CA, USA). Cells were collected and washed by cold phosphoate-buffered saline (PBS), then resuspended in Annexi-binding buffer and sustained with propidium iodide (PI) and FITC Annexin V. After incubating in the dark at room temperature for 15 min, cell suspensions were diluted by Annexin-binding buffer and analysed by BD LSRFotessa flow cytometry (SanJose, CA, USA) immediately. Data were acquired by BD FACSDiva software (SanJose, CA, USA).

### Cell proliferation assays

Analysis of cell proliferation was performed with cell counting kit-8 (Dojindo, Japan) assay. NVP-Bez235 and lenalidomide were purchased from Selleck (Huston, USA) and dissolved in DMSO. The treatment of BEZ235 was performed as 5nM, 10nM, 20nM and 40nM, while the working concentration of lenalidomide were 0.5 μM, 1 μM, 2 μM and 4 μM. Cells were seeded in 96-well plate at a concentration of 1 × 10^5^/mL. After 72 h, 10uL cell counting kit-8 were added to each well and incubated for 2 h. The absorbance at 450 nm was measured by a microplate reader. Growth inhibition was calculated by the formula (O.D absorbance of treatment group – O.D absorbance of blank)/(O.D absorbance of control group – O.D absorbance of blank) × 100 %. The synergetic effect of two drugs was measured by combination index (CI) using CalcuSyn software (Version 2.1). CI < 1 indicates the synergetic effect, CI = 1 means the additive effect and CI > 1 is regarded as antagonism.

### Immunobloting

NF-κB Pathway Sampler Kit, Akt, p-Akt (Ser 308), p-Akt (Thr 473), p21, p-CDK2, CDK2, cyclinA, cyclinD1, mTOR Pathway Sampler Kit, and Apoptosis sampler Kit were obtained from Cell Signaling Technology (Beverly, MA, USA). All the cell lines were treated with NVP-Bez235 and lenalidomide in single way or combination for 72 h. Bortezomib was purchased from Selleck (Huston, USA). Cells were performed with 5 nM Bortezomibe for 24 and 48 h, respectively. Then the samples in each group were collected and separated by standard SDS-PAGE gel electrophoresis and transferred to NC membrane. Blocked with 5 % non-fat milk supplemented in 0.1 % TBST, membrane was probed with primary antibodies at 4 °C overnight. After washed with TBST, membrane was incubated with HRP-conjugated secondary antibodies (Santa Cruz, CA) for one hour. The bands were detected and quantified in Image Lab software (Bio-Rad Laboratories, California, USA).

### Gene silencing with siRNA

NF-κB siRNA were purchased from Santa Cruz (CA, USA). Non-silencing siRNA was designed and synthesized as following: sense 5′-UUCUCCGAACGUGUCACGUTT-3′, antisense 5′-ACGUGACACGUUCGGAGAATT-3′. Cell lines were transiently transfected NF-κB siRNA and non-silencing siRNA by using LipofectAMINE2000 reagent (Invitrogen, Carlsbad, CA). After incubated for 8 h, lipid containing siRNA was removed and warmed complete medium was added. Cells were cotreated with NVP-Bez235 and lenalidomide for another 48 h following 24-h-transfection. Cells were collected for immunoblotting. Cell viability was measured when cells were transfected for 24 h followed with 48 h.

### Cell cycle assay

Each cell line was seeded in a 12-well plate exposing in drugs for 72 h. Then the cells were collected, centrifuged, washed with ice-cold PBS and resuspended with 70 % alcohol. After fixed in -20 °C for 12 h, the cells were washed and stained with PI and ribonuclease A (Beytime Instituted of Biotechnology, China) at room temperature for 30 min. Cell cycle data were assessed by BD LSRFortes flow cytometry (SanJose, CA, USA) and analyzed by Modfit LT 4.1 (Verity Software House, Topsham, USA).

### Mouse model

Six-weeks old nude female mice, purchased from Shanghai Laboratory Animal Center (Shanghai, China), were injected subcutaneously in the right flank with 1 × 10^7^ OCI-Ly10 cells in 100 μL saline. When the tumor volume reached to about 150–300 mm^3^, mice were randomly divided into 4 groups with 5 mice per group as follows: (I) control: intraperitoneally injected with saline; (II) NVP-Bez235: Bez235 20 mg/kg by oral gavage,dissoved in 10 % NMP (1-methyl-2-pyrrolidone)/PEG 300 90 %, every other day; (III) lenalidomide: intraperitoneally injected lenalidomide 10 mg/kg/day (diluted in 4 % DMSO, 96 % saline); (IV) combination: treatment of NVP-Bez235 with lenalidomide. The tumor volumes were measured every other day by vernier caliper for 28 days and calculated as V = a × b^2^/2(a, the length of tumor; b, the width of tumor). At the end of observation, mice were killed to remove the tumors. Then the samples were fixed in 4 % formalin and further performed to frozen section. Animal protocol was approved by the Animal Care and Use Committee of Ruijin Hospital affiliated to School of Medicine, Shanghai Jiao Tong University.

### Immunohistochemistry

Fixed slides obtained from samples of nude mice were incubated in 3 % H_2_O_2_ for 10 min to suppress endogenous peroxidase activity and then washed in PBS. Next, the slides were blocked with 1.5 % serum in PBS at room temperature for 30 min and subsequently in BSA for 20 min. Then the slides were incubated with primary antibodies p-NF-κB and Ki-67 (Santa Cruz, CA, USA) overnight. After washed for 15 min, the slides were added with secondary antibodies connected with biotin for half an hour and incubated with SABC (Boster, Wuhan, China). After incubating with a solution of DAB (Boster, Wuhan, China), the tissue slides must be checked under microscope to get appropriate staining. The final steps were counterstaining, dehydrating, clearing and mounting. The positive cells were counted using Image-Pro plus 6.0 (Media Cybernetics, USA).

### Terminal deoxytransferase-catalyzed DNA nick-end labeling (Tunel) assay

Frozen sections were performed with a terminal transferase recombinant kit (Roche, Indianapolis, IN, USA) under the guidelines of production information. Cells with green fluorescence were termed as the apoptosis section. Image-Pro plus 6.0 (Media Cybernetics, USA) were used to analyze the positive cells in each slide.

### Statistical analysis

All data in this study were analyzed with SPSS 17.0 software (Chicago, USA) and presented as mean ± standard deviation. Independent *t* test were used to determine the significance of difference between groups. IC_50_ value was analyzed by probit assay in SPSS. *P* < 0.05 was considered as statistically significance Overall survival was measured by Kaplan-Meier method. The drug interactions were stated according to Chou-Talalay method and isobologram analysis.

## Results

### Growth suppression of NVP-Bez235 and lenalidomide in ABC subtype of DLBCL cell lines

**Fig. 1:**
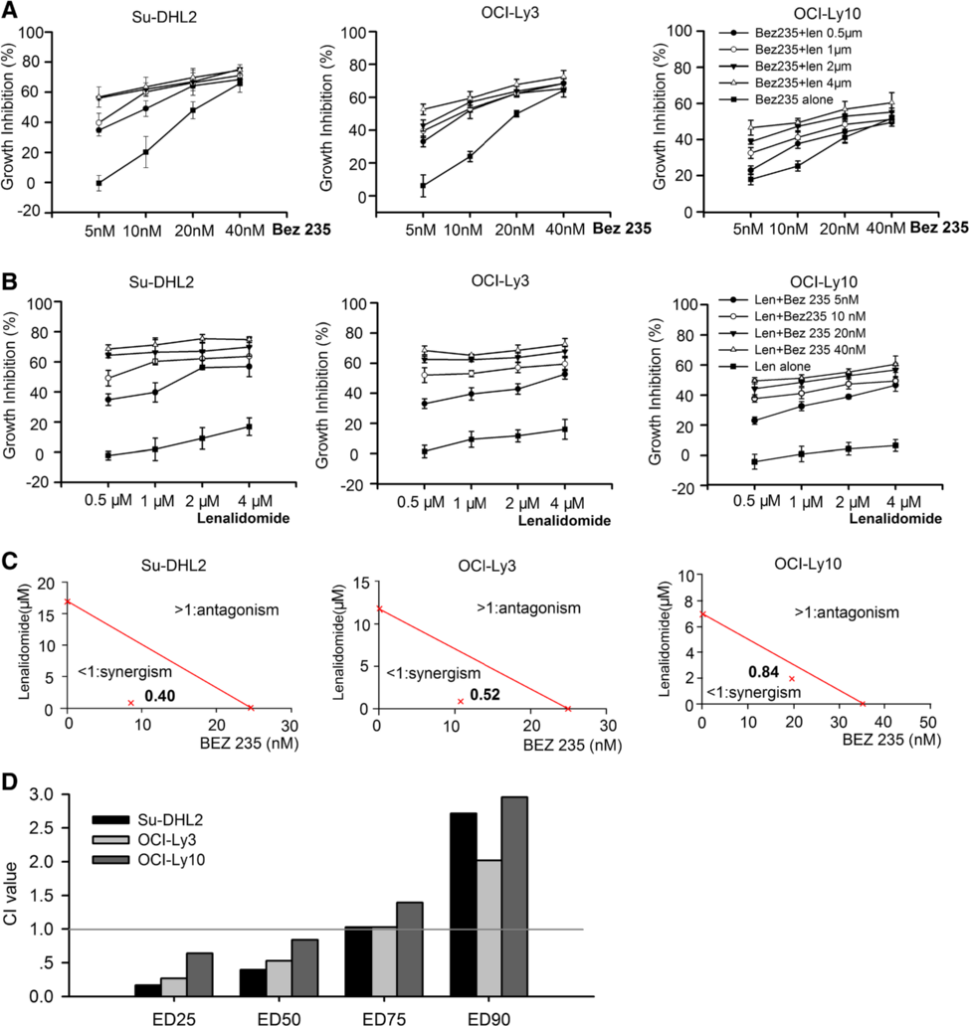
Combination effects of NVP-Bez235 and lenalidomide in proliferation inhibition. **a**, **b** Cell viability of Su-DHL2, OCI-Ly3, and OCI-Ly10 was measured by CCK-8 assay after treated with NVP-Bez235 and lenalidomide alone or in combination for 72 h. Drugs were conducted simultaneously at the indicated concentrations. **c** Isobologram analysis of agent combination at ED50 and CI values were calculated as well. **d** CI values of all cell lines at ED25, ED50, ED75 and ED90. The lower CI values indicated the stronger synergism when CI < 1, and higher CI values means the stronger antagonism when CI >1. Additive effect was designated with CI = 1

We choose 3 cell lines of ABC-DLBCL according to the public researches [6], which can partly represent the ABC-DLBCL characters. All cell lines represented dose-independent growth suppression when treated with NVP-Bez235 or lenalidomide for 72 h (Fig. 1a, b). The IC_50_ values of NVP-Bez235 were demonstrated in Table 1. The proliferation inhibition was remarkably increased when the dose of NVP-Bez235 was elevated. In contrast, lenalidomide showed moderate inhibition and even 4 μM lenalidomide cannot reach the IC_50_ in 72 h. As the maximum observed plasma concentration (C_max_) for patients treated with lenalidomide is 2.2 μM [23], we did not set higher doses when determining the cytotoxicity of lenalidimde in cell lines.

**Table 1:** IC_50_ values of Bez235 (5-40nM) in ABC- DLBCL cell lines for 72 h

	NVP-Bez235 (nM)
Su-DHL2	
IC_50_	27.76
CI 95 %	19.82–35.56
OCI-Ly3	
IC_50_	27.39
CI 95 %	22.92–31.86
OCI-Ly10	
IC_50_	36.86
CI 95 %	30.2–43.47

### Low dose of lenalidomide and NVP-Bez235 displays synergistic effect on ABC-DLBCL cell lines in vitro

All cell lines were treated with NVP-Bez235 (5nM, 10nM, 20nM and 40 nM) in combination with lenalidomide (0.5 μM, 1 μM, 2 μM, 4 μM) for 72 h to determine the combination effect. The growth inhibition was measured by CCK-8 assay (Fig. 1a, b), and further analyzed using isobologram analysis to validate the contributing effect of drug combination (Fig. 1c). The CI value was identified with 0.4 in Su-DHL2, 0.52 in OCI-Ly3 and 0.84 in OCI-Ly10 when the effective dose of both drugs inhibited the cell proliferation to 50 %. The low dose of lenalidomide (0.5-2 μM) substantially enhanced the proliferation suppression of NVP-Bez235 (5-20nM) with CI value ranging from 0.3 to 0.8 (CI < 1), indicating the synergistic effects of drug combination. In contrast, when lenalidomide reached a concentration of 4 μM concomitant with the concentration of NVP-Bez235 being at 40nM, the CI values in all cell lines were above 1, suggesting an antagonism effect of the drug combination (Table 2). As demonstrated in Fig. 1d, the CI value raised when the toxicity effects became higher. Specifically, the values were lower than 1 at ED25 and ED50, around 1 at ED75 and above 1 at ED90, which was in line with the proliferation inhibition showed in Fig. 1a, b. Therefore, the combination effect was correlated with the dose of components. Low dose of lenalidominde in combination with low dose of NVP-Bez235 represented a synergistic effect in cell proliferation, whereas high dose of two agents showed an antagonistic effect.

**Table 2:** Isobologram analysis of NVP-Bez235 in combination with lenalidomide

Cell lines	Bez235 (nM)	Len (μM)	CI
Su-DHL2	5	0.5	0.306
	10	1	0.391
	20	2	0.695
	40	4	1.193
OCI-Ly3	5	0.5	0.402
	10	1	0.464
	20	2	0.694
	40	4	1.062
OCI-Ly10	5	0.5	0.76
	10	1	0.607
	20	2	0.768
	40	4	1.165

### Combination of NVP-Bez235 and lenalidomide promote the apoptosis through caspase activation and Bcl-2 family regulation

**Fig. 2:**
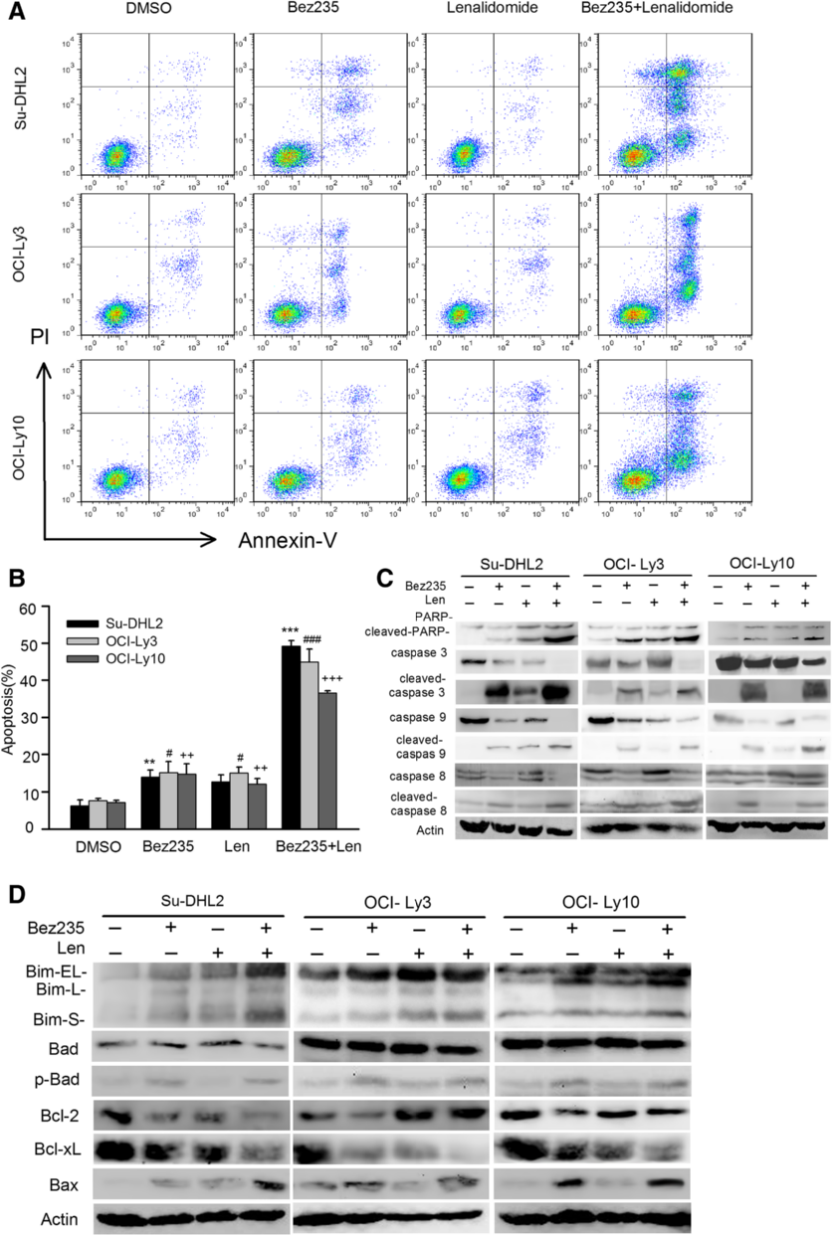
NVP-Bez235 combined with lenalidomide significantly increased the apoptosis in all cell lines. **a**, **b** Cell lines were simultaneously treated with NVP-BEZ235 (20nM) and lenalidomide (2 μM) for 72 h, analyzing apoptosis by Annexin-V/PI staining with *t* test statistic assay. (Mean ± S.D., *n* = 3, *, +, # *p* < 0.05, **, ++, ## *p* < 0.01, ***, +++, ### *p* < 0.005 compared with control group) (**c**) Cell lines were subjected to indicated treatments and protein lysates were performed with immunoblotting, incubating with PARP, caspase3, caspase9, caspase8 and their cleavage formate antibodies. **d** Cell extractions were conducted with Western Blot to detect Bcl-2 family members including Bax, Bad, p-Bad, Bim, Bcl-2 and Bcl-xL

To confirm whether the enhanced proliferation inhibition was caused by apoptosis, all cell lines were exposed to 20nM NVP-Bez235 and 2 μM lenalidomide either in single way or in combination. As is shown in Fig. 2a, b, apoptosis population was elevated from 36.5 % to 49.2 % under treatment of agent combination, which was significantly more than population of those treated with single agents (*p* < 0.05). OCI-Ly10 seemed less sensitive than OCI-Ly3 and Su-DHL2 (Fig. 2c). The apoptosis induced by drug combination was further confirmed by caspase family and bcl-2 family identification. Robust increased cleavage of caspase3 and poly-ADP-ribose polymerase (PARP) was observed in the combination group after 72 h. Subsequently, caspase 9 and caspase 8 were further examined to identify whether the effect was trigged by intrinsic or extrinsic signaling pathway. Our data showed that cleaved-caspase 9 significantly increased accompanied with the downregulation of total caspase 9 in the combination group. However, cleaved caspase 8 showed very modest increase by cotreatment compared with the NVP-Bez235 single treatment group, indicating the intrinsic pathway plays a predominant role in the apoptosis trigged by agent combination.

As the pivotal role of Bcl-2 family in the apoptosis intrinsic pathway, pro-apoptosis members (Bax, Bad, Bim) and anti-apopotosis members (Bcl-2 and Bcl-xL) were selected to determine the disturbance induced by single drug treatment or agents combination. Bim-EL and Bim-S were elevated remarkably by drug combination in all cell lines (Fig. 2d). The level of Bax was obviously upregulated by drug combination especially in Su-DHL2. However, we did not observe upregulation of p-Bad to a greater extent in combination group compared with NVP-Bez235 group. Among the anti-apoptosis molecules, Bcl-xL was remarkably downregulated by drug combination. Unlikely, Bcl-2 varied among cell lines. Agent combination induced Bcl-2 downregulation in Su-DHL2, upregulation in OCI-Ly3 and moderate change in OCI-Ly10. The results indicated that apoptosis trigged by drug combination was primarily mediated by intrinsic apoptosis pathway, of which the pro-apoptosis members overwhelmed the anti-apoptosis members.

### Akt and NF-κB pathway contribute to growth inhibition induced by drug combination

**Fig. 3:**
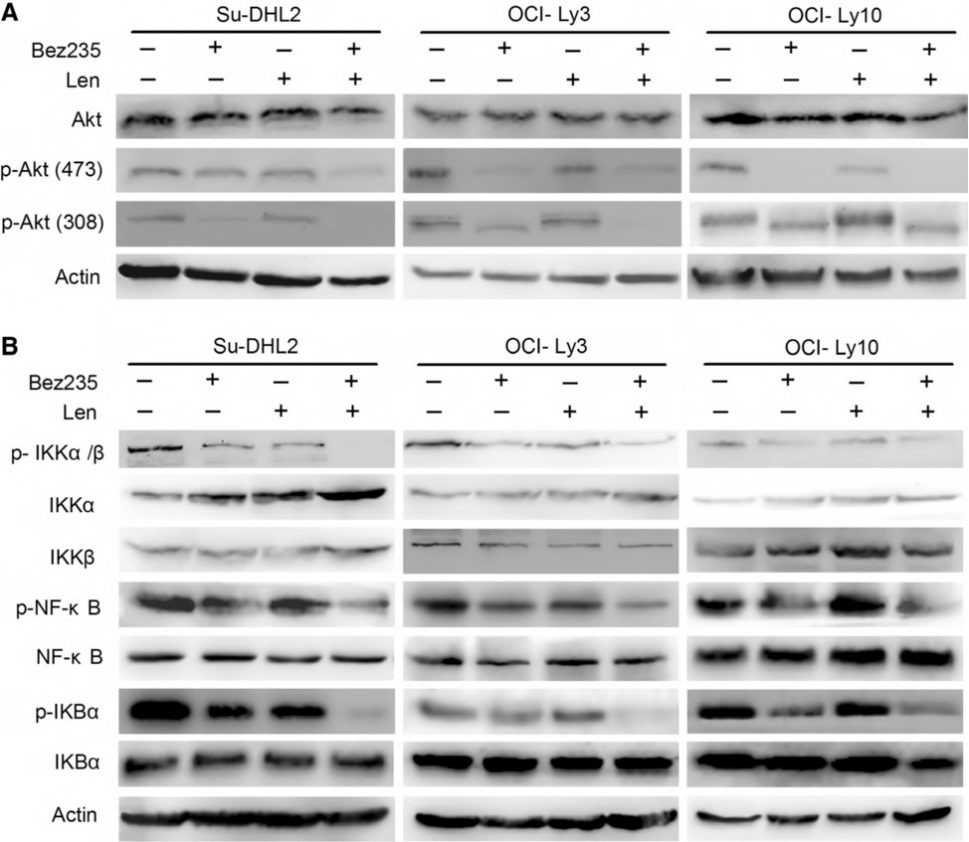
Synergistic inhibition of AKT and NF-κB pathway. **a** Cell lines were treated with NVP-Bez235 20nM and lenalidomide 2 μM alone or in combination for 72 h. Western blot analysis was performed to identify the expression of total Akt, p-Akt (Ser 473) and p-Akt (Thr308). **b** Western blot for Ikkα, Ikkβ, p-Ikkα/β, IΚBα, p-IΚBα, NF-κB and p-NF-κB

To further determine the mechanism of synergistic effect of drug combination, the expression of p-Akt (Ser473) and p-Akt (Thr308) was assessed by Western Blot. The results represented that combined NVP-Bez235 and lenalidomide significantly downregulated phosphorylation of Akt (Ser 473, Thr 308) in Su-DHL2 and OCI-Ly3 compared with the single agent treatment and control group, without affecting the total amount of Akt. In OCI-Ly10, phosphorylation of Akt (Ser 473) was totally blocked by NVP-Bez235 and combination components, while phosphorylation of Akt (Thr 308) only slightly reduced by Bez235 and drug combination (Fig. 3a).

**Fig. 4:**
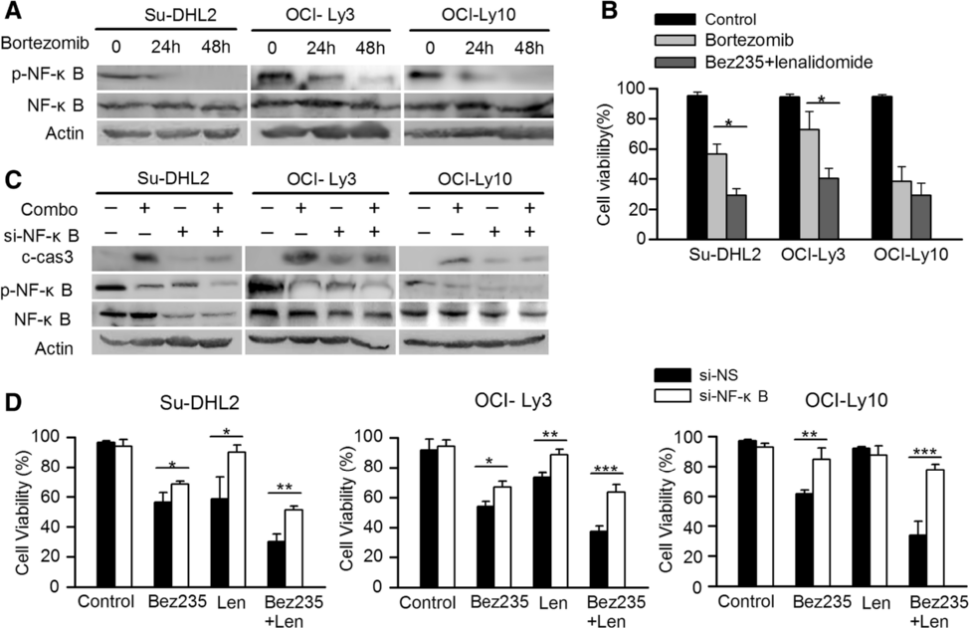
The role of NF-κB in apoptosis induced by NVP-Bez235 and lenalidomide. **a** Cell lines were exposed to 5nM bortezomib for 24 h and 48 h, respectively. Cell lysis were conducted with immunoblotting for p-NF-κB and NF-κB. **b** Bar graph was represented as mean ± S.D (*p < 0.05) to show the cell viability of cell lines treated with 5nM bortezomib and concomitant treatmemt of NVP-Bez235 (20nM) and lenalidomide (2 μM). **c** All cell lines were transiently transfected with siRNA against NF-κB (si-NF-κB) and nonsilencing siRNA (si-NS) respectively for 24 h and then exposed to NVP-Bez235 (20nM) plus lenalidomide (2 μM) for 48 h. Then cells were collected for Western blot. **d** Cells were transfected as (**c**) for 24 h and treated with compounds for another 48 h. Then CCK-8 assay was performed to calculate the cell viability. *p < 0.05, **p < 0.01, ***p < 0.005 compared with si-NS group

The results led us to further examine whether inhibition was similar in the NF-κB pathway. Our study revealed that the expression of p-IKKα/β, p-NF-κB and p-IΚBα were diminished to some extent after that the cell lines were exposed to single agent for 72 h, indicating using NVP-Bez235 or lenalidomide alone is not sufficient to block the NF-κB pathway. Unlikely, robust suppression of these molecules was observed in combined treatment of NVP-Bez235 and lenalidomide (Fig. 3b). In order to determine whether NF-κB plays a functional role in cotreatment anti-DLBCL activity, bortezomib, the first protesome inhibitor, was performed in the concentration at 5nM for 24 h and 48 h in all cell lines. Treatment with bortezomib represented significant downregulation of p-NF-κB with the cell viability reducing to 56.7 % and 72.8 % in Su-DHL2 and OCI-Ly3 respectively, which was not as strong as the combined treatment (*p* < 0.05) (Fig. 4a, b). However, the reduction of cell viability by agents combination seemed the same as bortezomib treatment in OCI-Ly10 after 72 h with no statistical significance (*p* > 0.05) (Fig. 4b), indicating the NF-κB pathway may play a more essential role in the apoptosis induced by NVP-Bez235 and lenalidomide in OCI-Ly10 than in Su-DHL2 and OCI-Ly3. To validate the role of NF-κB in drug induced apoptosis, Si-RNA of NF-κB was transfected to all cell lines. As is shown in Fig. 4c, knockdown of NF-κB protected cell lines from apoptosis with the reduction of caspase 3 cleavage compared with the non-silence siRNA group in all cell lines. Similarly, knockdown of NF-κB rendered cells more resistant to single agent or combination treatment (Fig. 4d). Taken together, we could conjecture that NF-κB pathway contributed to the apoptosis, but blocking NF-κB could not restore proliferation inhibition, suggesting other mechanisms may also contribute to cytotoxicity induced by drug combination.

### NVP-Bez235 and lenalidomide combination arrested cell-cycle in G0/G1 phase

**Fig. 5:**
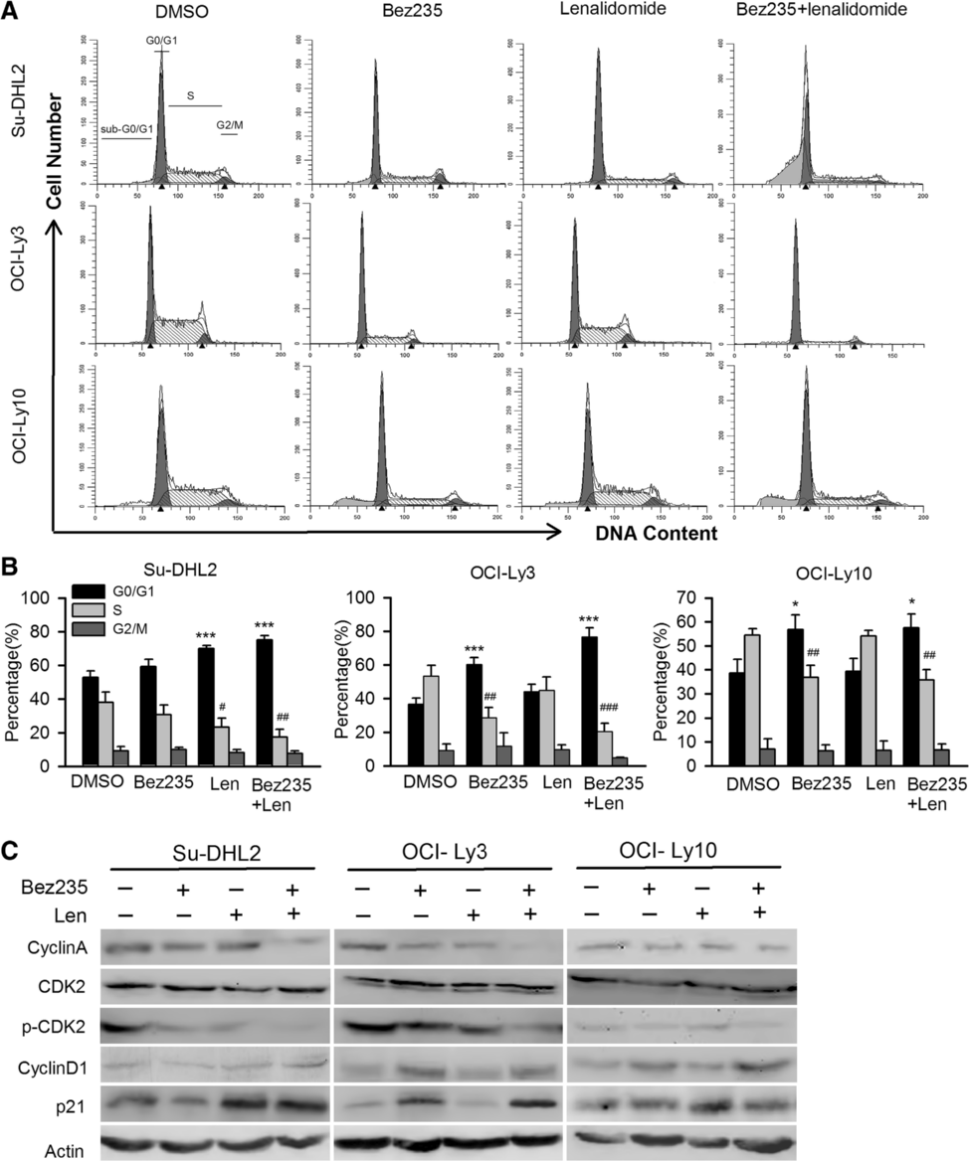
The effect of NVP-Bez235 and lenalidomide in cell cycle. **a** Cell lines were treated with components for 72 h, collected and fixed by 70 % ethanol for 24 h. Cell cycle assay was assessed with cytometry and Modfit LT. **b** The Bar graph illustrated the proportion of G0/G1 phase, S phase, G2/M phase of cell lines treated as mentioned above. Era bars indicated the standard deviation (*n* = 3). *, # *p* < 0.05, **, ## *p* < 0.01, ***, ### *p* < 0.005 compared with control group. **c** Western blot for cell lysis collected after treated with single agent or combination agents for 72 h

According to the results of proliferation inhibition above, cell-cycle detection was carried out under the treatment of indicated concentration to understand the synergism mechanism triggered by NVP-Bez235 and lenalidomide. The fraction of cells in S phase was significantly decreased by combination treatment (*p* = 0.009 in Su-DHL2, *p* = 0.002 in OCI-Ly3) compared with the DMSO group, accompanied by the increase of G0/G1 phase. There was no change in G2/M phase after cell cycle interfered with single agent or combination. In OCI-Ly10, drug combination did not increase G0/G1 phase or decrease S phase compared with NVP-Bez235 alone (Fig. 5a, b). Western blot was further performed to confirm the checkpoints associated with cell cycle. Combination of NVP-Bez235 and lenalidomide noticeably downregulated the expression of cyclinA and p-CDK2 in Su-DHL2 and OCI-Ly3, but not in OCI-Ly10 compared with the single agent treatment and control group (Fig. 5c), which was in accordance with the results of flow cytometry (Fig. 5a). Lenalidomide did not enhance the NVP-Bez235 function by upregulating cyclinD1. Meanwhile, lenalidomide significantly increased p21 expression in Su-DHL2, while NVP-Bez235 exerted similar effect in OCI-Ly3. By cotreatment, p21 was noticeably accumulated in both cell lines. In OCI-Ly10, p21 was not affected by drug treatment.

### Cotreatment of NVP-Bez235 and lenalidomide inhibit the mTOR pathway in vitro

**Fig. 6:**
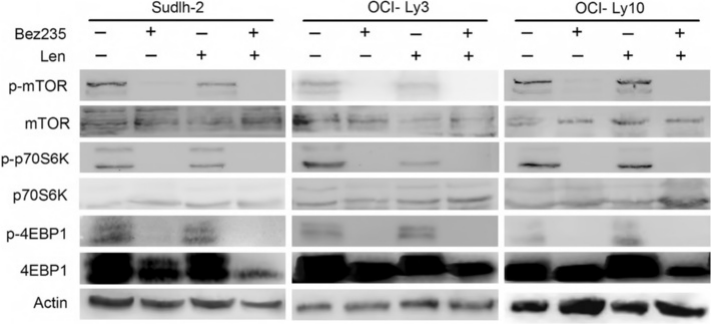
Combination treatment affected mTOR pathway. All cell lines were treated with NVP-Bez235 and lenalidomide for 72 h. Cell lysis was determined by immunoblotting to identify the protein mTOR, p-mTOR, p70S6K, p-p70S6K, 4EBP1, and p-4EBP1

As NVP-Bez235 was also the inhibitor of mTOR, we further determined whether the mTOR pathway contributed to synergism. As is shown in Fig. 6, mTOR pathway was totally abrogated by NVP-Bez235 combined with lenalidomide. The expression of total 4EBP1 was also reduced by cotreatment. These data suggested that mTOR pathway may also be involved in the synergetic effect in vitro.

### Combined low dose of NVP-Bez235 and lenalidomide suppressed tumor growth and prolonged the overall survival of the xenograft model of DLBCL

**Fig. 7:**
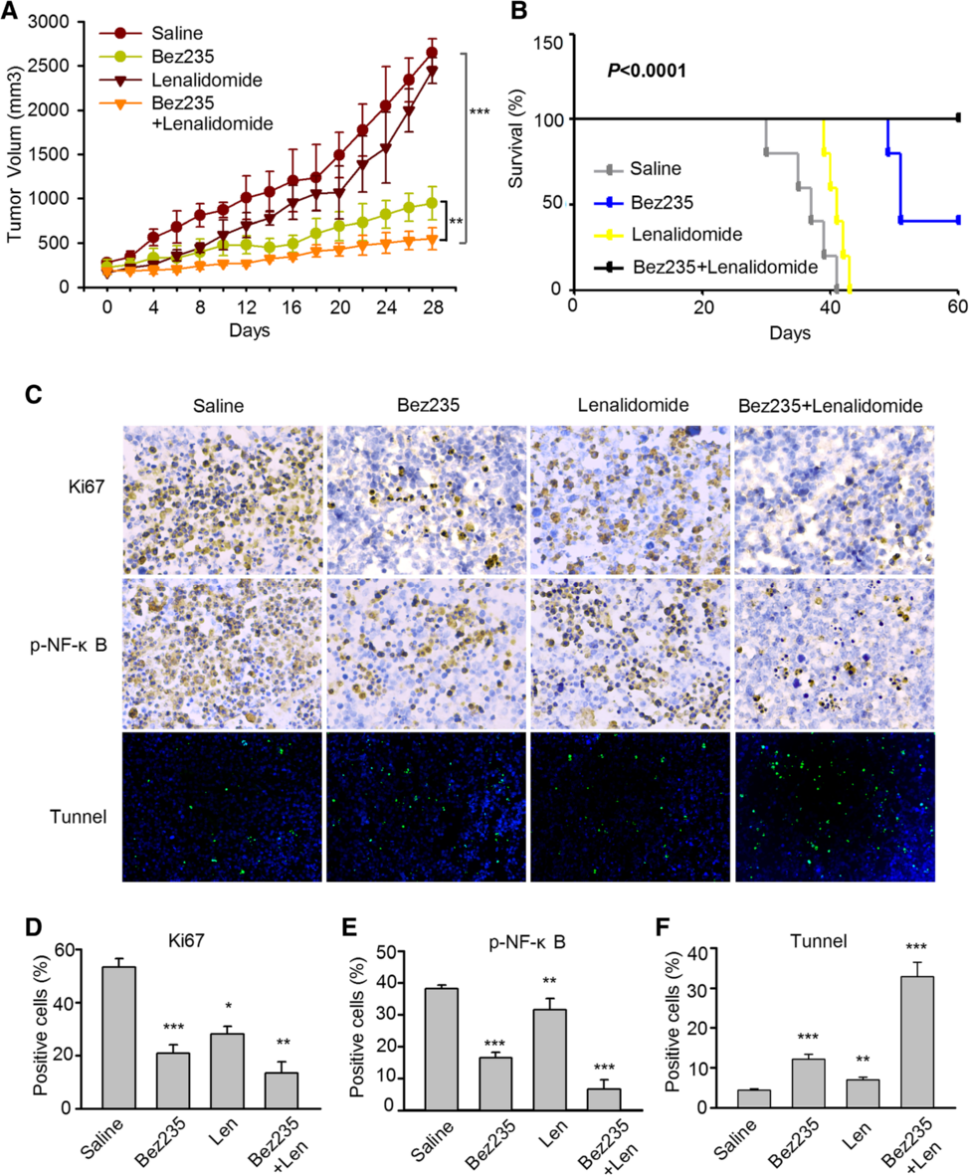
NVP-Bez235 plus lenalidomide dramatically inhibited tumor growth and prolonged the survival in a DLBCL xenograft mouse model (OCI-Ly10). **a** Mice in each cohort were treated with lenalidomide (10 mg/kg) every other day and NVP-Bez235 (20 mg/kg/day) alone or in combination. Tumor volumes were measured every other day for 28 days. At the end of observation, tumor growth was significantly delayed by combination treatment compared with control group (***p = 0.0003) and NVP-Bez235 treatment group (**p = 0.006). **b** Overall survival was prolonged by NVP-Bez235 and lenalidomide combination therapy compared with the control group and single agent group (*p* < 0.0001). **c** IHC to identify expression of Ki67, p- NF-κB of tumor specimen Tunnel assay was performed to examine the apoptosis in tissues. **d**, **e**, **f** Bar graph illustrate the proportion of positive cells showed in panel C. *p < 0.05, **p <0.01, *** *p* < 0.005 compared with the control group

The cytotoxic effect of NVP-Bez235 and lenalidmide alone or in combination were evaluated in the nude mice bearing OCI-Ly10 tumors. Mice were treated with NVP-Bez235 20 mg/kg in gavage, lenalidomide 10 mg/kg intraperitoneally or the combination. Tumor volumes in each group are shown in Fig. 7a. Daily treated with lenalidomide alone did not result in tumor growth delay (*p* > 0.05), whereas treatment of NVP-Bez235 alone significantly suppressed tumor growth compared with saline injection from 10 day onward (*p* < 0.001). Furthermore, lenalidomide enhanced the effect of tumor suppression of NVP-Bez235 when compared with NVP-Bez235 treatment (*p* < 0.01). All treatments were well tolerated with less body weight (<10 %) and no toxic death. Overall survival was prolonged after observation for 60 days with the median survival of 37 days in saline group, 57 days in NVP-Bez235 group and 41 days in lenalidomide group (*p* < 0.0001) (Fig. 7b). There was no death observed in the drug combination group at the end point of observation.

To confirm our studies of growth suppression mechanism, immunohistochemical analysis was conducted to identify the expression of Ki67 and p-NF-κB in each cohort. Combined treatment reduced the positive cells of Ki67 compared with control group (*p* < 0.01) and NVP-Bez235 group *(p* = 0.005) (Fig. 7d). While it seemed that lenalidomide moderately enhanced tumor suppression induced by NVP-Bez235 according to the comparison of Ki67 expression with NVP-Bez235 and cotreatment group (*p* = 0.04 < 0.05). Cotreatment of NVP-Bez235/lenalidomide also increased the apoptosis (*p* < 0.001)*,* revealed by Tunnel assay (Fig. 7f), which matched our previous studies in vitro. Although the positive cells stained with p-NF-κB were obviously reduced by NVP-Bez235 (*p* < 0.001), the drug combination did not enhance repression compared with the NVP-Bez235 group to a greater extent (*p* = 0.023 < 0.05) (Fig. 7e), which is not as effective as it did in vitro. Our results suggested that NVP-Bez235 combined with lenalidomide could remarkably delay the tumor growth by inducing apoptosis and inhibiting proliferation in vivo, but the mechanism of this suppression may be caused by multiple pathways including downregulation of p-NF-κB.

## Discussion

Combination strategy has represented lower toxicities and overcoming agent resistance in treatment of DLBCL. The oncogenetic molecular mechanisms involved in ABC-DLBCL were primarily initiated by BCR subunit activated mutations, as well as the *CARD11* and *MYD88* mutations, resulting in the constitutive activation of NF-κB [16, 24]. Meanwhile, constitutive PI3K signaling activation, resulted from the chronic BCR activation, was proved to support the viability of ABC-DLBCL [25]. For this reason, targeting proteins involved in these pathways is a rationale way in treating ABC-DLBCL. NVP-Bez235, a dual mTOR/PI3K inhibitor, was found to synergistically interact with BTK inhibitor Ibrutinib [26] and HDAC inhibitors [27]. In our study, we demonstrated that combination of low dose of NVP-Bez235 and lenalidomide played synergistic roles in killing ABC-DLBCL cells.

ABC-DLBCL cell lines showed different sensitivity to NVP-Bez235 and lenalidomide. Su-DHL2 and OCI-Ly3 were more vulnerable to single agent, but OCI-Ly10 showed a little resistant. Investigations of the direct cytotoxicity of lenalidmide in DLBCL [6, 28] have supported our findings. This difference also resulted in the variety in antiproliferation of combination treatment with different doses. The antagonistic effect appeared when drug doses escalated to higher level in all cell lines. Importantly, the synergistic effect was maintained when the concentrations of both drugs were well below clinical C_max_ [23, 29]. It was reported that NVP-Bez235 has no toxicity in normal CD34+ hematopoietic cells [27], and lenalidomide has limited long-term toxicity [30], suggesting the potential clinical application value of this combination strategy. We then examined the apoptosis induced by drug combination, and the results are in accordance with our previous findings. The apoptosis proportion of OCI-Ly10 was not as much as those of Su-DHL2 and OCI-Ly3. The difference of toxicity may be explained, at least partly, with the heterogeneity of DLBCL. OCI-Ly3 and OCI-Ly10 both maintain the *MYD88*-L256P mutation while Su-DHL2 gets the mutation of *MYD88*-S222R [16]. Meanwhile, mutation of *CARD11,* which induces the activation of IKK2 and NF-κB, also existed in OCI-Ly3 but not in OCI-Ly10 [24], and mutation of *TNFAIP3/A20* was observed in Su-DHL2 [31]. Collectively, the various mutation statues in each cell lines may be the underlying reasons of the various responses to single agent treatment or drug combination. In order to explain the different toxicity responses, we further examined the intrinsic pathway and extrinsic pathway, and found that apoptosis intrinsic pathway played a predominant role in the synergistic effect. To date, the complex process of apoptosis could be mainly categorized into intrinsic and extrinsic pathway, finally resulting in the DNA fragmentation and cell death. Stimulations from extracellular activated the transmembrane receptor and consequently triggered the activation of extrinsic initiator of caspase 8. In contrast, intrinsic pathway was initiated by mitochondrial events and resulted in the activation of caspase 9 through a more complex procedure. Finally, both extrinsic and intrinsic pathways would go through the execution pathway, which is considered as the end point of apoptosis. Caspase 3 is regarded as the most essential effector in the apoptosis execution pathway. Once it is activated by caspase 8 or caspase 9, the activated caspase 3 will further induce the cleavage of various subtracts such as PARP, which is a nuclear DNA-binding protein functioning as DNA breaks detector. The mitochondrial events of intrinsic pathway taken place in and controlled by Bcl-2 family, which can group into proapoptotic members and antiapoptotic members. At the initiation, Bim plays a crucial role [32]. Subsequently, oligomerization of Bak and Bax permeabiliaze the mitochondrial membrane and release SMAC and cytochrome c, further activating caspase9 and finally inducing apoptosis. The Bim up-regulation was required in the synergy of lenalidomide and NPI-0052 in MM [33], as well as in the contributing effect of NVP-Bez235 and HDAC inhibitor in DLBCL [27]. Here our results also showed that combined NVP-Bez235 and lenalidomide resulted in the accumulation of Bim-S, Bim-EL isoforms and Bax. Bad, a member of BH3-only protein, was regarded to exhibit the proapoptosis functions by directly binding and inhibiting anti-apoptotic member Bcl-2 and was reported to be associated with the DBLCL tumorigenesis [34]. We did not find stronger phosphorylation of p-Bad in the combination treatment, suggesting other members may exert more important efforts in the apoptosis. We next identified the dysregulation of anti-apoptotic members including Bcl-2 and Bcl-xL. The Bcl-xL expression was significantly reduced by drug combination. However, the Bcl-2 expression varied among the cell lines. One reason may partly explain the chaos of Bcl-2. The oncogenic function of Bcl-2 was first reported as its overexpression could promote the c-myc driven proliferation [35]. Patients who suffered with the “double-hit” DLBCL, representing the translocation of *MYC* with the arrangements of *BCL2, BCL6* or *CCND1*, often turn out to be with poor prognosis [36]. *C-MYC* amplification was found in OCI-Ly3 but not in OCI-Ly10 [37], and there was no report on the *C-MYC* status in Su-DHL2. These led us to assume that the diverse of MYC expression in cell lines causes the Bcl-2 variation in different cell lines. We did not examine the change of anti-apoptotic member Mcl-1 induced by drug combination. To our knowledge, Mcl-1 was elevated in ABC-DLBCL [38], and many pre-clinical studies have reported its necessity in drug-induced apoptosis of DLBCL [27, 39, 40]. Altogether, our results demonstrated that the drug combination induced the apoptosis through the intrinsic pathway by elevating the cleaved-caspase 9, Bim and Bax, as well as downregulating the expression of Bcl-xL.

The mechanism involved in the synergetic effect was further depicted by examination of Akt and NF-κB pathway. Since lenalidomide was reported to exert its immunomodulatory function through PI3K/Akt pathway in CLL [11], we found that lenalidomide only slightly dephosphorylated p-Akt (Ser 473) in OCI-Ly10 and p-Akt (Thr 308) in Su-DHL2, which in line with the study of lenalidomide cytotoxicity in MCL [41]. Robust downregulation of p-Akt (Ser 473) and p-Akt (Thr 308) was observed in Su-DHL2 and OCI-ly3 by co-treatment, indicating the importance of PI3K/Akt in the synergetic effect. The activation of NF-κB was well considered to support the viability of ABC-DLBCL. Recurrent somatic mutations resulted in the activity of NF-κB varied among cell lines, of which MYD88-L256P got higher NF-κB activity than MYD88-S222R [16]. Specifically, the expression of NF-κB, p-p65 and p52 was predicted to be a poor prognosis factor of DLBCL in some studies [42, 43], as well as the p-IΚBα in ABC-DLBCL [44], but the expression of Rel was reported to be associated with superior clinical outcome [45]. Tracing the validation of NF-κB pathway in prognosis, function of NF-κB was estimated to cooperate with *FOXP1* and *BLIMP1* in ABC-DLBCL [46, 47]. Abundant researches indicated that NF-κB monitored the cell life and death through regulating apoptosis program in a caspase-independent way [48]. Regarded as an anti-apoptosis transcription factor, NF-κB could induce a number of gene expressions, of which the production acts as the apoptosis inhibitor. C-IAPs and c-FLIP are well established NF-κB-regulated proteins. Besides, NF-κB was also found to modulate the Bcl-2 family and pro-/anti-apoptosis pathways through TRAF1 and TRAP2 [49]. Owing to the important role of NF-κB in cell fate, members involved in the NF-κB pathway were further detected. We found that NVP-Bez235 and lenalidomide treatment also substantially downregulated NF-κB pathway. However, we did not assess whether suppression of Akt activity was involved in the regulation of NF-κB, but it seemed that PI3K could only directly affect high level of nuclear NF-κB in DLBCL cell lines [25]. In contrast, other studies indicated that NF-κB was controlled by PI3K [50, 51]. In addition, as NVP-Bez235 also inhibited the mTOR pathway, whether NF-κB is independent with mTOR pathway requires further study. Of note, our confirmation of the role of NF-κB in synergetic effect also implied that the mechanism could not be simply explained by the inhibition of Akt and NF-κB pathways, so there must be other biological impacts contributing to this effect.

Cell cycle arrest has been observed in the anti-NHL and anti-CLL activity of PI3K inhibitor as well as lenalidomide [12, 41, 52–54]. In our study, sharp decrease of S phase was observed in Su-DHL2 and OCI-Ly3 cell lines with apparently increase of G0/G1 phase after exposed in both NVP-Bez235 and lenalidomide, suggesting the cell cycle arrest contributed to the combined cytotoxicity. Furthermore, in view of importance of CDK2/cyclinA in G1/S phase transition [55], abrogation of cyclinA and dephosphorylation of p-CDK2 in Su-DHL2 and OCI-Ly3 by cotreatment may contribute to the ultimate cell death. Indeed, deregulation of CDK2 and CDK4 genes has been found to be associated with the transformation process of the transformed DLBCL [56], additionally, *CDKN2A* deletion was predicted for poor prognosis in DLBCL [57], suggesting the important role of CDK2 in pathogenesis of DLBCL. It was known that p21 and p27 normally arrest cell cycle by suppressing the activity of CDKs, but we did not examine the expression of p27. p21 could be upregulated by lenalidomide [12], which is in line with our studies, and was even associated with prognosis, for example, p53 + p27 + p21-p16- and low p27 expression was suggested with poor clinical outcomes and shorter survivals [58, 59]. CyclinD1 was accumulated by NVP-Bez235/lenalidomide cotreatment, which is in contrast with the results of lenalidomide in MCL [41]. The overexpression of cyclinD1 in DLBCL, accounting for 1.5–15 % [60–62], showed no prognosis value [63], indicating the cyclinD1 may not be as important as cyclinA/CDK2. The mechanism by which Akt or NF-κB inhibition diminish cyclinA or p-CDK2 in our study is unclear, but Chiron et al reported that PI3K inhibitors induced cell cycle arrest by downregulating CDK4/6 [64], and NF-κB was reported to be associated with cell cycle regulation [65].

Finally, our in vivo studies demonstrated that concomitant treatment of NVP-Bez235 and lenalidomide exerted potential antitumor activitity with well tolerant doses. Specifically, although lenalidomide single treatment did not show anti-DLBCL activity in vivo, it significantly enhanced NVP-Bez235 antitumor potency by delaying the tumor growth and prolonging the overall survival. Moreover, the apoptosis and growth inhibition with cotreatment in vitro could also be detected in tumor tissues. Unlikely, NVP-Bez235/lenalidomide cotreatment did not inhibit the phosphorylation of p-NF-κB to a great extent as it did in in vitro studies, suggesting that the mechanism of antitumor activity in intact animals may be more complex than the mechanisms in vitro.

## Conclusions

Collectively, our study demonstrated that NVP-Bez235 combined with lenalidomide effectively inhibited cell proliferation, induced apoptosis and cell cycle arrest in ABC-DLBCL cell lines. Moreover, it was also active in the DLBCL xenograft model. The present findings also suggest that the mechanisms are likely associated with regulation of Akt and NF-κB pathway, inducing the apoptosis through upregulation of Bim, Bax and downregulation of Bcl-xL in all cell lines. Lenalidomide did not enhance the potency of NVP-Bez235 in OCI-Ly10 by inhibiting G1/S transition withincreasing p21 expression, downregulating cyclinA and dephophorylating p-CDK2. Notably, NF-κB may also be related to the drug resistance of DLBCL [66]. Therefore, our present studies raise the possibility of a novel strategy to treat ABC-DLBCL patients by using the combination of NVP-Bez235 and lenalidomide.
